# Editorial: Lifestyle modifications to manage migraine

**DOI:** 10.3389/fneur.2022.966424

**Published:** 2022-08-29

**Authors:** Yohannes W. Woldeamanuel, Surya Shrivastava, Marta Vila-Pueyo

**Affiliations:** ^1^Division of Headache & Facial Pain, Department of Neurology & Neurological Sciences, Stanford University School of Medicine, Stanford, CA, United States; ^2^Independent Researcher, Los Angeles, CA, United States; ^3^Headache and Neurological Pain Research Group, Department of Medicine, Vall d'Hebron Research Institute, Universitat Autònoma de Barcelona, Barcelona, Spain

**Keywords:** migraine, lifestyle and behavior, headache, regular lifestyle behavior, lifestyle medicine

We read with interest the Research Topic articles on “*Lifestyle Modifications to Manage Migraine*.” Here, we present summaries and insights on regular lifestyle behavior (RLB)—followed by an overview of possible molecular mechanisms implicated in the modulation of migraine through RLB.

Raucci et al.'s review on lifestyle-based childhood migraine management highlighted the landmark 24-week comparative effectiveness RCT (randomized controlled trial) in which 61% of children on placebo (involving RLB, regular sleep, hydration, mealtime, exercise) achieved a 50% monthly reduction in headache frequency compared to 55 and 52% on daily topiramate and amitriptyline, respectively (Raucci et al.) (1, 2). RLB preempted drug-induced adverse effects (Raucci et al.) (1, 2). RLB education in adolescence [migraine onset peak age (Raucci et al.) (3, 4)] helps inculcate anti-migraine behavioral habits. Dehydration [a migraine precipitant (5, 6)] is common in children in North America and Europe (Raucci et al.) (7, 8). Unfavorable lifestyle-related habits that increase migraine burden e.g., sedentariness/obesity, screen time, smoking/alcohol/psychoactive substance use, stress (e.g., school-related bullying), and caffeine/cola consumption are rising in adolescents (Raucci et al.). Addressing lifestyle factors is central in pediatric headache management (Raucci et al.).

Lisicki et al. conducted a two-phase real-world study to understand dietary migraine triggers. The first phase cross-sectional study examined whether food/drink avoidance differs between people with and without migraine. Although 64.3% of people with migraine reported avoiding a food/drink type, there was no significant group difference in consumption between those with and without migraine. In a follow-up 2-month prospective diary study, chocolate, wine, sweeteners, and cheese were frequently consumed before migraine onset. Food cravings and decreased appetite were reported before a migraine attack. The authors suggested that consumption of “attack-triggering” food items may be a migraine prodrome rather than a cause. However, published RCTs demonstrate the efficacy of elimination diets in migraine (9, 10). Intraindividual changes and absolute or partial (additive or potentiating) (11) triggers may confound the potentially bidirectional migraine-diet relationship (12) complex. Ensuring a balanced regular meal and regarding diet as just one component of lifestyle-based migraine management is generally recommended.

Grozeva et al. took advantage of the mandatory COVID-19 lockdowns to examine the impact of lifestyle changes (e.g., sleep, work) on migraine in pre-post longitudinal cohorts. During the first COVID-19 lockdown which lasted 6–8 weeks, there was a reduction in migraine burden. However, during the second lockdown, the migraine burden returned to its basal/higher levels. The authors posited that sudden short-term (6–8 weeks) lifestyle changes may benefit migraine patients. This article indicates how observational studies and RCTs complement each other. RCTs may not always be ideal to study complex lifestyle behaviors (sleep, exercise) due to known challenges e.g., self-selection bias, fidelity, blinding. Observational studies also have their share of problems e.g., confounders, endogeneity, selection bias. A recent large-scale RCT proved that *H. pylori* eradication reduces the risk of gastric cancer (13)—validating what is already known in observational studies. Do we need to wait for further evidence from multiple RCTs [or a natural disaster, as the authors phrased it (Grozeva et al.)] before we recommend adopting lifestyle changes (e.g., regular sleep, stress coping skills) shown by observational studies to reduce migraine burden? Not really.

Rivera-Mancilla et al. examined the relationship between 3 common chronic conditions i.e., migraine (14.4% prevalence), obesity (13%), and diabetes mellitus (9.3%). The authors elucidated that the migraine-obesity relationship may be bidirectional due to shared lifestyle and biological risk factors, seen in clinicoepidemiological and interventional studies. RLB (e.g., exercise) is linked to the central and peripheral nervous system in migraine, obesity, and diabetes (Rivera-Mancilla et al.). Obesity can result from low physical activity following migraine disability and anti-migraine drug-induced weight gain (e.g., beta-blockers, antidepressants, anticonvulsants, calcium channel blockers) (Rivera-Mancilla et al.). The diabetes-migraine relationship is not clear (Rivera-Mancilla et al.). CGRP levels are high in migraine and obesity, while low in type-2 diabetes (Rivera-Mancilla et al.). These results do not add up considering that obesity (risk for migraine) causes diabetes. Does migraine (or migraine medications) mediate or moderate the risk obesity poses to diabetes/insulin resistance? Topiramate is the only anti-migraine drug resulting in fat loss among non-diabetic migraine patients without high BMI (Rivera-Mancilla et al.). Does topiramate have the same effect in diabetic and obese migraine patients? Weight loss interventions (behavioral, bariatric surgery) lead to migraine reduction (Rivera-Mancilla et al.). Given that CGRP modulates insulin release (Rivera-Mancilla et al.) (14), can long-term CGRP blockage in migraine patients influence diabetes (15)? Compared to obesity, migraine with aura has a stronger association with major cardiovascular or cerebrovascular diseases (16). More studies are needed to clarify the ischemia risk from CGRP blockade (17–19), particularly considering the complex migraine-obesity-diabetes triumvirate relationship.

## Possible molecular mechanisms of regular lifestyle behavior for migraine control

RLB is attributed to adherence to three circadian-related aspects of daily life i.e., sleep-wake, mealtime, and exercise (20). Disrupted sleep, irregular mealtime, and sedentariness worsen migraine outcomes (20). These three arms of lifestyle can alter the epigenetic status of the genome, including modifications of histones, the DNA methylation status of specific genomic regions, and/or the expression of non-coding RNAs (such as microRNAs): three mechanisms that up- or down- regulate gene expression (21–24). A previous review summarizes the evidence showing the effects that sleep can have in modulating these epigenetic factors (22) and other studies have shown similar impacts for exercise (25) and regular mealtime (26, 27).

**Figure 1:**
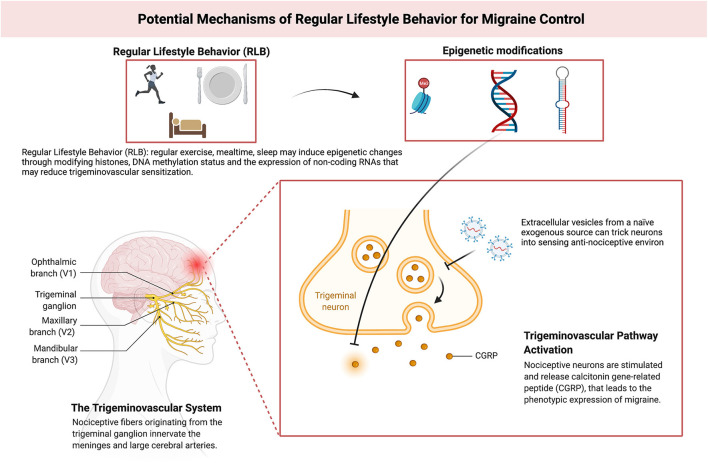
Model depicting putative mechanisms of Regular Lifestyle Behavior (RLB) for migraine control. Adapted from “The role of CGRP and the trigeminal system in migraine pathophysiology”, by BioRender.com (2022). Retrieved from https://app.Biorender.com/biorender-templates.

RLB adherence can be speculated to enhance migraine outcomes by modifying neuronal, epigenetic, and genetic factors. Deviation from RLB might induce macro and micro-environment changes, affecting neuronal epigenetics, that may get propagated through inter-cellular communication leading to neurovascular sensitization and neuroinflammation. Extra-cellular vesicles (EV) are harbingers of inter-cellular communication and they could be implicated in this process (28, 29). Pro-migraine inter-cellular information could be substituted by enriching the micro-environment with EV from a naïve exogenous source (e.g., stem cells) to trick cells into sensing the anti-migraine environment (30, 31). This might reverse epigenetic modifications caused by RLB deviation, leading to migraine control (Figure 1). Exogenous EV are systemically short-lived (28), their anti-migraine effect could be ephemeral against chronic RLB deviation. Stem cell-derived EV can best serve as adjunct migraine therapy along with RLB—enhancing RLB maintenance.

Besides epigenetics, other RLB-related molecular mechanisms in migraine include the strong link between the pathophysiology of headache and sleep (32); including several brainstem nuclei playing a role in both entities, the hypothalamus implicated both in regulating circadian rhythms and generating migraine attacks (33), and the modulation of sleep and headache by the same orexinergic systems (34). Another key point is the existing molecular link between appetite and migraine pathophysiology, where orexins also play a crucial role (35). Orexins are two hypothalamic neuropeptides linked to appetite regulation, wakefulness, and the perception and integration of pain (36), hence one could hypothesize whether these molecules may be involved in the increased susceptibility of migraine during RLB disruption.

## Conclusion

This Research Topic articles provide an overview of how lifestyle modifications may affect migraine. Future studies on this topic can improve our understanding of this process and may unravel the precise molecular mechanisms involved, which eventually could lead to the development of new therapeutic targets.

## Author contributions

YW drafted the first version of the manuscript. SS and MV-P prepared the molecular mechanisms section. All authors revised the final version, approved the manuscript and figure, and provided critical feedback and helped shape the research.

## Funding

YW received research funding from the NINDS (National Institute of Neurological Disorders and Stroke), NIH (National Institutes of Health) (1K01NS124911-01). MV-P is a recipient of a Sara Borrell contract from the Instituto de Salud Carlos III, Ministerio de Ciencia e Innovación, Spain (CD20/00019).

## Conflict of interest

The authors declare that the research was conducted in the absence of any commercial or financial relationships that could be construed as a potential conflict of interest.

## Publisher's note

All claims expressed in this article are solely those of the authors and do not necessarily represent those of their affiliated organizations, or those of the publisher, the editors and the reviewers. Any product that may be evaluated in this article, or claim that may be made by its manufacturer, is not guaranteed or endorsed by the publisher.
